# Supplementary material for: A Critical View of the Application of the APEX Software (Aqueous Photochemistry of Environmentally-Occurring Xenobiotics) to Predict Photoreaction Kinetics in Surface Freshwaters
Source: Molecules. 2019 Dec 18;25(1):9. doi: 10.3390/molecules25010009 (PMC7017383; doi:10.3390/molecules25010009)
Supplement: Supplementary file 1 [file molecules-25-00009-s001.zip › 3.2.4_gcc-4.4.0.zip!/3.2.4_gcc-4.4.0/3.2.4_gcc-4.4.0/doc/octave/html/interpreter/Calling-Functions.html]

Calling Functions - Untitled


Next: Arithmetic Ops,
Previous: Index Expressions,
Up: Expressions


---

### 8.2 Calling Functions

A function is a name for a particular calculation. Because it has
a name, you can ask for it by name at any point in the program. For
example, the function `sqrt` computes the square root of a number.

A fixed set of functions are built-in, which means they are
available in every Octave program. The `sqrt` function is one of
these. In addition, you can define your own functions.
See Functions and Scripts, for information about how to do this.

The way to use a function is with a function call expression,
which consists of the function name followed by a list of
arguments in parentheses. The arguments are expressions which give
the raw materials for the calculation that the function will do. When
there is more than one argument, they are separated by commas. If there
are no arguments, you can omit the parentheses, but it is a good idea to
include them anyway, to clearly indicate that a function call was
intended. Here are some examples:

```
     sqrt (x^2 + y^2)      # One argument
     ones (n, m)           # Two arguments
     rand ()               # No arguments
```

Each function expects a particular number of arguments. For example, the
`sqrt` function must be called with a single argument, the number
to take the square root of:

```
     sqrt (argument)
```

Some of the built-in functions take a variable number of arguments,
depending on the particular usage, and their behavior is different
depending on the number of arguments supplied.

Like every other expression, the function call has a value, which is
computed by the function based on the arguments you give it. In this
example, the value of `sqrt (`argument`)` is the square root of
the argument. A function can also have side effects, such as assigning
the values of certain variables or doing input or output operations.

Unlike most languages, functions in Octave may return multiple values.
For example, the following statement

```
     [u, s, v] = svd (a)
```

computes the singular value decomposition of the matrix `a` and
assigns the three result matrices to `u`, `s`, and `v`.

The left side of a multiple assignment expression is itself a list of
expressions, and is allowed to be a list of variable names or index
expressions. See also Index Expressions, and Assignment Ops.

- Call by Value- Recursion
